# Validation of the Sleep Quality Questionnaire among senior students in Benin City, Nigeria

**DOI:** 10.4102/sajpsychiatry.v28i0.1875

**Published:** 2022-12-14

**Authors:** Oluyemi Akanni, Anthony Olashore, Olaide Koleoso

**Affiliations:** 1 Clinical Services, Federal Neuropsychiatric Hospital, Benin City, Nigeria; 2 Department of Psychiatry, Faculty of Medicine, University of Botswana, Gaborone, Botswana; 3 Department of Psychology, Faculty of Medicine, University of Benin Teaching Hospital, Benin City, Nigeria

**Keywords:** insomnia, Nigeria, psychometrics, Sleep Quality Questionnaire, students

## Abstract

**Background:**

The Sleep Quality Questionnaire (SQQ) is a short and easy-to-understand resourceful tool for measuring poor sleep quality; however, it remains to be validated.

**Aim:**

The focus of this study is to determine its reliability and validity among Nigerian adolescents.

**Setting:**

Four gender-mixed schools within Benin City, Nigeria were selected to participate in the study.

**Methods:**

Questionnaires containing the SQQ, the Sleep Condition Indicator (SCI), which is a validated scale, and the Hospital Anxiety and Depression Scale (HADS) from 377 students selected from the junior and senior secondary school students were analysed.

**Results:**

The mean age calculated was 14.78 years for 174 male and 203 female students. Cronbach’s alpha for the SQQ scale was 0.70. It correlated strongly with the SCI (rho = 0.93, *p* = 0.00), weakly with the HADS depression (rho = -0.19, *p* < 0.01) and anxiety (rho = -0.30, *p* < 0.01), but had no association with gender. Factor analysis revealed three factors with an eigenvalue greater than 1. Factors 1–3 accounted for 31.78%, 15.16% and 11.26% of the variance, respectively, totalling 58.2%. The reliability of each of the three factors was as follows: factor 1, 0.75; factor 2, 0.47; and factor 3, 0.62. The SQQ demonstrated good specificity and sensitivity at a cut-off point of 18.

**Conclusion:**

It is therefore recommended that the SQQ be included in the screening of sleep-related problems in adolescents, both in the primary and secondary care settings in Nigeria.

**Contribution:**

The research shows that the Sleep Quality Questionnaire is both a reliable and valid screening tool among adolescents in Nigeria. Its brevity and simplicity further promote its use in clinical and non-clinical settings.

## Introduction

Sleep is a universal process that is a naturally recurring physiological phenomenon essential for rest, repair, learning and development.^1^ A good sleep quality in the adolescent age group has been described as a total sleep time of 85% or more of the total time in bed, commencing sleep not later than half an hour while attempting sleep, waking up not more than once during the night and being able to recommence sleep within 15 min of an initial awakening.^2^ When it is disrupted, it could have both physical and psychological impacts, and for school-going adolescents, it could cause an impairment in academic performance.^3^

Insomnia is the commonest form of sleep disruption among adolescents.^4^ A community study of more than 1000 adolescents found insomnia in one out of 10 of them.^5^ It is therefore important to be able to accurately screen the disorder among adolescents because the occurrence is high.

Many instruments have been designed to identify insomnia among adolescents; a commonly used and reliable one is the Pittsburgh Sleep Quality Index. However, it takes a total of 15 min to complete and score.^6,7^ Other available tools can accurately serve the same purpose and at the same time be administered within a shorter time. In a setting such as Nigeria, where time is a constraint,^8^ such a scale becomes invaluable. Two tools, the Sleep Quality Questionnaire (SQQ) and Sleep Condition Indicator (SCI), are therefore of interest in being very short and understandable for use in detecting poor sleep quality among Nigerian adolescents.^9^

The two instruments have the same items, except that the SQQ has an additional item. Also, they differ in that the SQQ is yet to be validated whereas the SCI has been previously validated.^9^ The SCI has thus gained widespread use more than the SQQ, and it was found to correlate well with some health indices, such as depression and anxiety.^9,10^ In addition, the SCI was found to have gender-referenced values^10^ and has been further reduced to a two-item version for use in general practice.^11^ Thus, the authors aimed to determine the reliability of the SQQ and the concurrent, convergent validity of the instrument. Consequently, they embarked on validating the SQQ among Nigerian adolescents by correlating it with the SCI and mental health parameters such as anxiety and depression. Moreover, they sought to investigate any association between the SQQ and gender, and also establish smaller factors or constructs within the instrument by conducting an exploratory factor analysis (EFA).

## Research methods and design

### Study setting and population

The study was conducted in Benin City, the capital of Edo State, located in the South-South geopolitical zone of Nigeria. It was carried out among secondary school adolescents who were expected to understand the English language enough to fill the questionnaire appropriately. Within the city are schools either owned by the government (public) or individuals (private). The secondary school system is divided into junior and senior levels, and each is a three-year program. Therefore, the study population consisted of students from both levels of secondary education.

### Sampling method

Sampling was based on convenience. Four gender-mixed schools consisting of two public and private schools were selected from the schools in the city. The selection was purposefully made to ensure the representation of public and private schools. The students were thereafter randomly selected from both junior and senior secondary levels of the chosen schools. In total, 420 students were sampled, which is well beyond the recommended ratio of 10 cases for each item to be factor analysed.^12^

### Data collection

The authors used a questionnaire which consisted of four sections.

#### Sociodemographic characteristics section

This was used to collect information such as the gender, age, family structure, school type, religion and ethnicity of the students. It was purposefully designed and structured to meet the objectives of the study.

#### The Sleep Condition Indicator

It is an eight-item self-report scale used to assess one’s sleep quality over a one month time interval.^9^ A few of the items are: ‘Thinking about the past month, to what extent has a poor sleep affected your mood, energy, or relationships?’ and ‘How many nights a week do you have a problem with your sleep?’ It can be completed and scored within 3 min – 5 min. It is a five-point Likert scale (0–4), which, when added, gives a possible score range from 0 to 32. The lower the score, the more severe the sleep problem is, whereas higher scores indicate that sleep is good. A score of 16 and below is highly suggestive of problems with one’s sleep. Therefore, this score was adopted as the cut-off point in this study.

#### The Sleep Quality Questionnaire

The Sleep Quality Questionnaire is a nine-item scale that was yet to be validated.^13^ It is similar to SCI except for an additional item three, which reads: ‘If your final wake-up time occurs before you intend to wake up, how much earlier is this?’ The scale is also scored on a five-point scale (0–4), and the responses are added together to give a total score. Possible scores range from 0 to 36. Because of variations that may exist in health measure outcomes as a result of culture and language,^14^ the instrument was subject to scrutiny by the authors to ensure clarity for the target test-taker. The scale required no language or cross-cultural modification because the reading level was rated low and language expression was simple to understand.

#### The Hospital Anxiety Depression Scale

This scale is used to screen for anxiety or depression in clinical and nonclinical populations. It consists of seven depression and anxiety items each and has been validated for use in Nigeria.^15^ A higher score on each scale indicates higher psychological distress.

### Procedure

Before the collection of data, approval was obtained from the Ministry of Education and the authorities of the schools involved. Informed consent was obtained from the participants and their parents or teachers if the participants were below the age of 18 years. Only the students who could give consent or assent were selected. Two adequately trained research assistants were employed for the collection of data. The training entailed understanding the questionnaire’s content enough to assist the students when necessary. The administration of the questionnaire was done in the classroom, mostly during the break period. During the questionnaire administration, the students understood the wording of the questionnaire and needed no form of assistance in filling it out. Data were collected between 22 January 2018 and 02 February 2018.

### Data analysis

The data were imputed into the Statistical Package for the Social Sciences (SPSS) version 22 (IBM Corporation, Armonk, New York, United States) for analysis. The participants’ characteristics were summarised using descriptive statistics. A normality testing using the Kolmogorov–Smirnov test was performed on the sleep variable. The reliability of the SQQ scale was assessed using Cronbach’s alpha coefficients. Convergent validity was verified by correlating it with the SCI and the anxiety and depression scores of the Hospital Anxiety and Depression Scale (HADS), using a Spearman’s correlation, whereas the association with gender was ascertained by an independent *t*-test. Exploratory factor analysis was performed to delineate the underlying factor structure of the SQQ in the sample. Before the factor analysis, Bartlett’s tests of sphericity and the Kaiser–Meyer–Olkin index were carried out to assess the data’s suitability for factor analysis. Principal components analysis was used to extract the factors, followed by an oblique rotation of factors using oblimin rotation to aid the interpretation of the components extracted. The number of factors to be retained was guided by the scree plot and the Kaiser’s criterion of eigenvalues above 1. The level of significance was set at *p* < 0.05 a priori.

### Ethical considerations

Prior to the collection of data, approval was obtained from the Ministry of Education’s Research and Statistics Department (STT1465T/188) and the authorities of the schools involved. Informed consent was obtained from the participants and their parents and/or teachers if the participants were below the age of 18 years.

## Result

### Sociodemography of the participants

In total, 377 copies of the questionnaire out of the 420 filled ones, which is 90%, were analysed. The participants comprised 174 (46.2%) male and 203 (53.8%) female students. The ages ranged between 10 and 21 years, with a mean of 14.78 years (standard deviation [s.d.] = 1.83) and a median of 15.00 years. They consisted of 189 students from public schools and 183 (49.2%) from private schools. Other characteristics of the participants are indicated in Table 1.

**TABLE 1:** Participants’ characteristics.

Variables	Frequency	%
**Gender**		
Male	174	46.2
Female	203	53.8
**School †**		
Public	189	50.8
Private	183	49.2
**Ethnicity †**		
Bini	159	43.9
Non-Bini	203	56.1
**Family †**		
Monogamy	294	80.5
Polygamy	71	19.5
**Religion †**		
Christianity	359	95.7
Others	16	4.3

Note: Median age is 15 years.

† , Missing data.

### Reliability of the Sleep Quality Questionnaire

The median score of the SQQ scale was 27, with the scores ranging between 3 and 36. There were no differences in mean SQQ scores across gender (*t* = 0.58, *p* = 0.56). Cronbach’s alpha for the scale was moderately strong at 0.70, and the range of α-if-item-deleted was 0.65–0.70. The mean inter-item correlation was 0.22, which is acceptable for a scale with items less than 10. Furthermore, the McDonald’s omega point coefficient measured 0.71 (confidence interval [CI]: 0.65–076), which is acceptable.

### Concurrent validity and relationship with psychological domains

A normality testing using the Kolmogorov–Smirnov test was performed on the sleep variable, and it came out not normally distributed. Hence, a nonparametric test using Spearman’s correlations was applied to determine the relationship between the variables. The concurrent validity of the nine-item SQQ was established by correlating the scores with the validated eight-item SCI. The result showed a significant positive correlation with a strong degree of association (rho = 0.93, *p* = 0.00), thus making it acceptable for the study. On the other hand, the analysis of SQQ revealed a negative but weak relationship with the depression (rho = -0.19, *p* < 0.01) and anxiety (rho = -0.30, *p* < 0.01) component of the HADS.

### Exploratory factor structure of the Sleep Quality Questionnaire

The assessment carried out for the suitability of the scale for factor analysis confirmed the factorability of the matrix. Bartlett’s test of sphericity was significant (*p* = 0.00), and also the Kaiser–Meyer–Olkin index yielded an adequate value of 0.77. Three factors appeared to emerge on inspecting the scree plot, and this was confirmed following a principal component analysis with eigenvalues greater than 1. Factors 1–3 accounted for 31.78%, 15.16% and 11.26% of the variance, respectively, totalling 58.2%. The reliability of each of the three factors was as follows: factor 1, 0.75; factor 2, 0.47; and factor 3, 0.62. Following a direct oblimin rotation, a simple structure of each of the variables loading strongly on only one component was reported on the pattern and structure matrix (Table 2). Going by the items which loaded together, the factors were named factor 1: Insomnia consequences (on the mood, relationships, concentration and productivity, generally); factor 2: Insomnia type (early, middle, late); factor 3: Insomnia characteristics (frequency, severity, duration). The lowest communality was above the acceptable 0.3 value, indicating that all the items fit well with the other items.

**TABLE 2:** Pattern matrix for principal components analysis with Oblimin rotation of three-factor solution.

Component items	1	2	3
7	0.853†	−0.034	−0.038
6	0.753†	−0.078	0.169
8	0.750†	0.104	0.077
2	−0.007	0.728†	0.061
1	0.215	0.696†	−2.740
3	−0.181	0.618†	0.214
4	0.020	0.099	0.745†
9	0.127	−0.146	0.742†
5	0.213	0.284	0.495†

† , factors loaded together.

### Diagnostic accuracy of Sleep Quality Questionnaire

To standardise the scale for diagnostic use, the sensitivity, specificity, positive predictive value, and negative predictive value of the various cut-off scores of SQQ were determined. Table 3 shows, how they were measured against the SCI standardised cut-off score of 16. A score of 18 was found to be the ideal cut-off score to screen for cases of insomnia using the SQQ because it achieved a sensitivity of 89.1% and specificity of 97.9% at that point.

**TABLE 3:** Psychometric properties of Sleep Quality Questionnaire in screening for insomnia.

Cut-off of SQQ	Specificity	Sensitivity	PPV	NPV
14	91.4	100.0	35.4	100.0
15	92.7	100.0	45.8	100.0
16	94.6	100.0	60.4	100.0
17	95.9	94.4	70.8	99.4
**18**	**97.9**	**89.1**	**85.4**	**98.5**
19	98.8	81.5	91.7	97.0
20	99.4	70.8	95.8	94.2

Note: The significance of the boldface are factors loaded together.

SQQ, sleep quality questionnaire; PPV, positive predictive value; NPV, negative predictive value.

## Discussion

This study is an effort to validate a screening tool for insomnia problems among Nigerian adolescents. Sleep problems are common among children and adolescents in Nigeria, and they are associated with physical and mental health indices.^16^ One way to address sleep problems among these people is to devise a simple, inexpensive but reliable way of diagnosing it in the community clinics, where many of these youth present. A brief and valid self-report scale can help screen patients with sleep problems, even in primary care settings. This study set out to validate such an instrument among Nigerian adolescents.

The validation of the instrument was found to require no adaptation for use among the sample. There was no need for translating the instrument because the participants were literate enough to comprehend the English language. However, because adaptation has been suggested for scales measuring health-related variables when they are to be applied in another cultural setting,^14^ the authors scrutinised the instrument to ensure there were no cultural, idiomatic or semantic expressions that could lead to a misinterpretation of the items of the instruments. The failure of the students during the filling of the questionnaire to express any difficulty or challenge corroborated this.

The present study found that the SQQ correlated well with the SCI, a well-established and validated eight-item instrument, thus suggesting the possibility of giving similar results when used in the study population. Compared to the SCI’s ability to predict depression and anxiety, the SQQ showed a weaker correlation with depression on the depressive component of the HADS, possibly because of the nature of our sample. However, its correlation with anxiety on the HADS was modest and similar to that of the SCI (*r* = -0.400).^9^ This may lend credence to the usefulness of the instrument in predicting important mental health outcomes. Furthermore, there were no gender differences in the SQQ scores, unlike what was reported for the SCI,^10^ although a similar result has been recorded elsewhere with other sleep quality measuring scales.^17^ This finding implies that the SQQ screening tool requires no gender distinction for referencing.

This study indicates acceptable reliability of the SQQ among Nigerian adolescents in terms of internal consistency. These data support the performance of the SQQ as a measure of sleep problems. This finding is comparable to the validation of the Sleep Difficulty subscale by Kato,^17^ who reported a Cronbach’s alpha of 0.74 among 1400 Japanese students. The alpha coefficient obtained in this report is regarded as reliable because all the α-if-item-deleted were in the range of 0.65–0.70, which is satisfactory. Also, the calculation of omega alongside a CI reflects a more accurate degree of confidence in the consistency of the scale.

The structure matrix of SQQ was established by the EFA because there was no proposed theory of any underlying factor. The criteria applied for extraction are considered dependable, although the scree plot has some subjectivity to its interpretation. With items fewer than 30, an average extracted communality of approximately 0.60 and an adequate sample size, the conditions required for the Kaiser criterion to be reliable were met.^18^ Additionally, an oblique rotation was chosen because it produces more accurate results for research involving human behaviours.^19^

The outcome of the analysis supports three structures, which were most strongly related to the underlying construct meeting the criteria for poor sleep quality. These three subscales are namely insomnia type, its characteristics and the consequences. Each has three items; for example, the items (1, 2 and 3) that measure the ‘insomnia type’ indicated if the insomnia is early, middle or late. Whereas the items (4, 5 and 9) on the ‘insomnia characteristics’ indicated the frequency, severity and duration of poor sleep, the ‘insomnia consequences’ subscale has items (6, 7 and 8) that measure the impact of insomnia on the mood and relationships, concentration and productivity and in general. Notably, two of these subscales have inadequate alpha reliabilities, which creates a challenge for their independent use. It is thus advisable for the instrument to be used as a whole, because the composite internal consistency score is acceptable.

When the SQQ was compared with the standard SCI, it showed reasonable accuracy in identifying insomnia cases. Although at a cut-off score of 17, the SQQ could have achieved better specificity, it drastically reduced the positive predictive value to a much lower value of 70.8. However, when the SQQ is compared with the SCI at a cut-off score of 18, it showed very good specificity and diagnostic ability in detecting probable insomnia in the sampled population.

This study has some limitations to be noted. Firstly, the selection of the schools was based on convenience; a multistage sampling would have been a better sampling technique to provide a representative sample. Secondly, the stability of the SQQ was not determined because it was a cross-sectional study, although a test-retest would have solved this challenge. Despite these limitations, the SQQ is a resourceful and suitable tool for detecting poor sleep quality in the adolescent population in Nigeria.

## Conclusion

The Sleep Quality Questionnaire is considered a simple, reliable and accurate instrument suitable for detecting poor sleep quality in the adolescent population in Nigeria. It demonstrates good specificity and sensitivity at a cut-off point of 18. In addition, this screening tool can assist mental health professionals, such as psychiatrists, clinicalpsychologists and therapists operating at various healthcare centres in Nigeria. Therefore, it is recommended that the SQQ be included in the screening of insomnia in adolescents, both in Nigeria’s primary and secondary care settings.
